# Factors influencing intercultural competences of hospital workers for multicultural patients in South Korea

**DOI:** 10.5116/ijme.6667.2270

**Published:** 2024-06-21

**Authors:** Bo La Kim, Hyojin Bae, Heejin Choi, Myongsoon Sung

**Affiliations:** 1 College of Liberal Arts and Cross-Disciplinary Studies, University of Seoul, South Korea; 2 Research Team, Soonchunhyang University Gumi Hospital, South Korea; 3 Department of Pediatrics, Soonchunhyang University Gumi Hospital, South Korea

**Keywords:** Multicultural patients, hospital workers, professional development, intercultural competence, communication skills

## Abstract

**Objectives:**

This study aims to assess the intercultural competence of general hospital workers in South Korea by examining their understanding of cultural diversity in healthcare and to identify factors influencing their intercultural competence.

**Methods:**

A cross-institutional survey was conducted with 439 participants from four South Korean general hospitals, employing inferential statistics such as one-way Analysis of Variance, Mann-Whitney U, and Kruskal-Wallis test followed by post-hoc, and multiple linear regression analyses.

**Results:**

While 85% (n = 362) of participants acknowledged the significance of multiculturalism in Korean society, only 11% (n = 49) felt competent in treating multicultural patients. Additionally, 72% (n = 315) experienced significant linguistic difficulties in medical communication. Multiple regression analysis identified advanced English competency, multicultural training experiences, and peer support with organizational awareness of multicultural importance as significant positive contributors to intercultural competence.

**Conclusions:**

Despite recognizing the importance of multiculturalism, general hospital workers face significant language barriers and low self-efficacy in providing care to multicultural patients. To address these challenges, hospitals should designate resident translators for culturally appropriate communication. Furthermore, a tri-tiered training approach is proposed to enhance the five domains of intercultural competence among general hospital workers in Korea, including overarching multicultural training, occupation-specific courses, and long-term managerial programs aimed at managing cultural diversity effectively in healthcare settings.

## Introduction

In 2019, a Vietnamese woman who visited a private obstetrics and gynecology clinic in the Republic of Korea (Korea) experienced an abortion without obtaining her informed consent due to language barrier, leading to legal action against the medical staff.^1^ She had initially sought nutritional supplementation. Another case is involved a Muslim patient declining a life-sustaining treatment plan for malignant lymphoma; healthcare providers were uncertain if the refusal was based on religion or the cost of treatment.^2^ These incidents underscore the challenges of intercultural medical communication in Korea. About 86.3% of foreign or multicultural pregnant women in the country report significant communication pressure in medical facilities.^3^ These cases highlight the critical need for Korean hospital workers to navigate diverse linguistic, cultural, and religious backgrounds while providing medical care.

The growing significance of multiculturalism in Korea since the 1990s is closely linked to the increasing number of foreign workers and multicultural families.^4^^,^^5^ In Korea, multicultural populations can be broadly categorized into three groups: multicultural families with one native Korean and one foreigner, immigrant populations as foreigners, and North Korean defectors.^6^ Recent data from the Korea Statistical Information Service^7^ and the Ministry of Employment and Labor^8^ reveal that Korea is home to approximately 385,219 multicultural families (1,119,267 individuals) and about 368,893 foreign workers with E-9 (non-professional employment) and H-2 (work and visit) visas. These demographic shifts not only accentuate the diverse composition of Korean society but also signify the country’s increasing integration into the global community, a response to challenges such as low birth rates and workforce shortages.

Despite the societal progress, Korea’s historical homogeneity of over 5,000 years has resulted in some individuals harboring xenophobic attitudes and concealed anxieties or aversions towards foreigners.^9^^,^^10^ The United Nations Committee on the Elimination of Racial Discrimination has recognized that the emphasis on ethnic purity homogeneity in Korea, often framed as ‘pure blood vs. mixed blood,’ could hinder the promotion of ethnic diversity and inadvertently foster intolerance and prejudice.^11^ This historical backdrop is further reflected in contemporary issues, such as discriminatory COVID-19 policies mandating tests for non-Koreans in certain regions.^12^^-^^14^ These policies have sparked debates about human rights and discriminatory practices on foreign and multicultural populations. In this context, a pressing question arises: Are Korean hospital workers adequately prepared to treat patients from other ethnic backgrounds than their own?

### Hospital systems in Korea

In Korea, the healthcare system is structured into primary hospitals, secondary medical institutions that provide specialized treatments, and tertiary medical institutions that offer highly specialized services, advanced medical equipment, and teams of professional medical personnel.^15^ Tertiary medical institutions are further divided into general hospitals and upper-tier facilities. General hospitals are required to have more than 100 beds and a minimum of seven medical specialties for up to 300 beds. If the bed count exceeds 300, the hospital must offer more than nine medical specialties.^15^ The Ministry of Health and Welfare designates upper-tier general hospitals from among general hospitals every three years.^16^ This process aims to optimize medical resources, provide high-quality services for severe diseases, and establish an efficient medical delivery system.

Additionally, Medical Law, Act No. 17787 (2021)^17^ defines various healthcare provider occupations, including doctors, nurses, nursing assistants, medical technicians (e.g., clinical pathologists, radiologists, physical therapists, and dental hygienists), and pharmacists. Hospital service workers (e.g., administration personnel, coordinators, receptionists, medical assistants, and medical information managers) and hospital operation workers (e.g., facility managers, safety managers, transportation teams, and hospital assistants) are categorized and titled based on each hospital’s preferences. Investigating the intercultural competences of general hospital workers becomes imperative due to their crucial roles in providing comprehensive medical care, including emergency, end-of-life, or life-threatening situations.

### Previous studies and current research gaps

Intercultural competence is defined as an individual’s ability or proficiency to effectively interact, communicate, and collaborate with people from different cultural backgrounds. Common components of intercultural competences include cultural awareness, knowledge, sensitivity, and communication skills.^18^ Building on this, the importance of intercultural competence also spans various domains in Korea, significantly contributing to the preservation of basic human rights, such as education,^19^^-^^21^ social welfare,^22^^-^^24^ and law enforcement.^25^^, ^^26^

In the healthcare domain, several studies have explored aspects related to multiculturalism. These investigations have examined factors impacting medical satisfaction in multicultural populations,^27^ assessed the intercultural competences of nursing staff and nursing students,^28^^-^^31^ explored medical students’ attitudes towards a multicultural society,^32^ investigated cultural knowledge and sensitivity among health and welfare college students,^33^ scrutinized dental care-related situations,^34^^,^^35^ and evaluated healthcare providers’ stress when treating multiethnic patients.^36^

**Figure 1:**
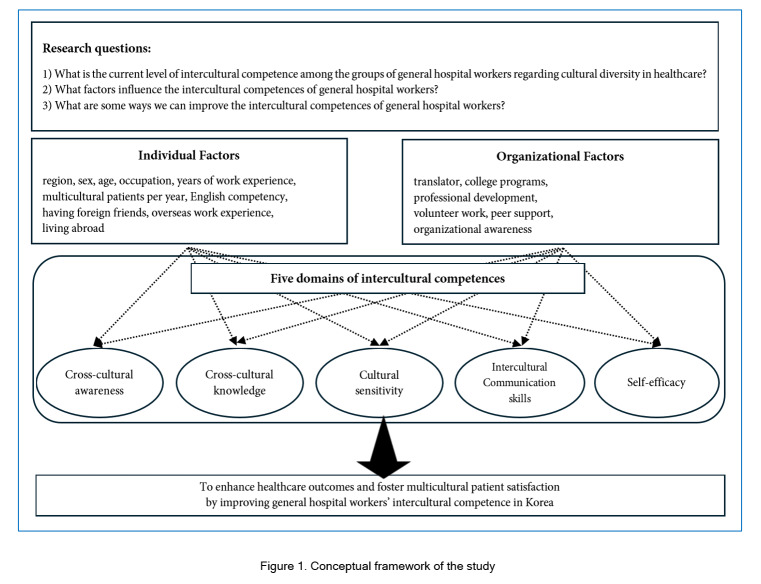
Conceptual framework of the study

Moreover, effective intercultural communication is considered one of the essential virtues for healthcare professionals worldwide. Research on healthcare providers’ intercultural communication skills has been conducted in countries such as the United States, the United Kingdom, and New Zealand.^37^^-^^39^ As healthcare systems increasingly serve diverse patient populations from various cultural backgrounds, the ability of hospital workers to understand and adapt to these differences is considered crucial to improve healthcare outcomes.^40^ Hospital workers who can navigate these diverse cultural and linguistic landscapes provide better healthcare,^41^ enhance patient satisfaction and ensure equitable healthcare access.^42^ In an era of increasing globalization and multiculturalism, developing intercultural communication skills becomes more vital for healthcare workers.^38^Figure 1 demonstrates the conceptual framework of this study. While previous studies have provided valuable insights into specific occupational roles and fields regarding intercultural competences, they often present a narrow focus. For instance, general and upper-tier hospitals in Korea are equipped with elevated expertise and resources, housing highly trained professionals capable of diagnosing and treating intricate medical conditions, including traumatic cases.^43^ However, little is known about the holistic intercultural competence of general hospital workers, who manage both medical and non-medical responsibilities, potentially adding complexity to the medical care process.^44^ Consequently, there is a vital need for a comprehensive examination of general hospitals and their workers. Thus, this study aims to assess the current level of intercultural competence among general hospital workers in Korea’s healthcare settings, identify the factors influencing their intercultural competences, and propose strategies to enhance their overall intercultural competence in addressing cultural diversity within healthcare environments

## Methods

### Study design and participants

This study employed a convenience sampling method due to practical considerations such as accessibility to participants and time constraints, aiming to efficiently gather data from a diverse groups of healthcare workers across different regions in Korea. This approach allowed individuals to self-select and volunteer for participation, ensuring that the sample consisted of motivated and willing participants. The survey was distributed for two weeks from November 7, 2022, among healthcare workers, including healthcare providers, medical technicians, pharmacists, nursing assistants, and hospital service and operation workers located from four regions in Korea, one was in the capital, three were in suburban areas. The required sample size for this cross-institutional study was initially set at 262 participants. To account for potential missing data, about 50% was added to the sample size.

A total of 443 responses were received. However, after identifying and excluding four duplicate responses based on mobile numbers, the final dataset for analysis comprised 439 participants. The majority of the participants were healthcare providers, constituting 65.8% (n=289), primarily doctors and nurses. Following this, 18.7% (n = 82) were hospital service workers, 7.3% (n=32) were medical technicians, 5.9% (n = 23) were nursing assistants, and 1.1% (n = 5) each were pharmacists and hospital operation workers, respectively.

The demographic and background information of the sample revealed that 23% (n=99) were males, while 77% (n = 340) were females. In terms of English competency, variability was observed, with 18% (n=79) having no competency, 62% (n=274) classified as beginners, 17% (n=76) as intermediate, and two percent (n=10) as advanced. Regarding the annual count of foreign and multicultural patients treated, 22.8% (n=100) reported none, 47.2% (n=207) handled one to nine cases, 17.8% (n=65) managed 10 to 19 cases, and 15.3% (n = 67) attended to more than 20 cases (Table 1).

Moreover, the participants’ demographics indicate a diverse range of multicultural experiences. Table 2 provides a summary of the participants’ responses to the multiple-response questions. Notably, 51.5% (n= 226) indicated no prior multicultural experiences. Approximately 45.1% (n=198) reported having foreign friends, while 5.7% (n=25) had work experience abroad, and 11.2% (n=49) had lived abroad for over six months. In terms of multicultural training experience, 74.9% (n=329) had not received any specific training, 13.7% (n= 60) had training during undergraduate education, 3.4% (n=15) had gained professional development in intercultural communication, and 12.8% (n=56) had participated in volunteer activities. As for organizational settings and resources, 22.3% (n=98) reported having no support, 30.1% (n=132) were encouraged by their colleagues to participate in multicultural-related events, 44.9% (n=197) stated that their organization recognized the importance of cultural inclusion, and 49.4% (n=217) had access to hospital translators.

The study received ethical approval from the Soonchunhyang University Gumi Hospital Institutional Review Board (IRB). The authors confirm that all the methods were carried out in accordance with relevant guidelines and regulations.

**Table 1:** The summary of the participants’ demographic and background information

Category (Abb.) (Question numbers)		N=439	%
Regions (Q0)			
	Seoul	82	18.7
	Bucheon	146	33.3
	Cheonan	124	28.2
	Gumi	87	19.8
Sex (Q1)			
	Male	99	22.6
	Female	340	77.4
Age (Q2)			
	20~29	89	20.3
	30~39	127	28.9
	40~49	135	30.8
	≥ 50	88	20.0
Occupation (Q3 & Q3-1)			
	Hospital service workers	82	18.7
	Nursing assistants	26	5.9
	Healthcare providers	289	65.8
	Medical technicians	32	7.3
	Pharmacists	5	1.1
	Hospital operation workers	5	1.1
Years of work experience (Q4)			
	1~4 years	107	24.4
	5~9 years	80	18.2
	10~19 years	109	24.8
	≥ 20 years	143	32.6
English competency (Q6)			
	None	79	18.0
	Beginner	274	62.4
	Intermediate	76	17.3
	Advanced	10	2.3
Multicultural patients attended to per year (Q7)			
	0	100	22.8
	1~9 patients	207	47.2
	10~19 patients	65	14.8
	≥ 20 patients	67	15.3

**Table 2:** The summary of the participants’ responses for the multiple response questions

Multiple response survey questions		N	%
Multicultural experiences (Q5)			
	No experience	226	51.5
	Have foreign friends	198	45.1
	Overseas work experience	25	5.7
	Lived abroad ≥ 6 months	49	11.2
Multicultural training experience (Q13)			
	No experience	329	74.9
	Had in undergraduate course	60	13.7
	Had professional development	15	3.4
	Volunteer work	56	12.8
Organizational settings and resources (Q20)			
	No support	98	22.3
	Peer support	132	30.1
	Organizational awareness of multicultural importance	197	44.9
	Translator(s)	217	49.4

### Data collection methods

We conducted a cross-institutional survey involving hospital workers from two upper-tier hospitals (Bucheon and Cheonan) and two general hospitals (Seoul and Gumi) over a two-week period starting on November 7th, 2022. Participants were informed via internal emails, which included the Google Survey link and Quick Response code. The survey targeted individuals who had worked at the hospital for over a year and were familiar with its organizational settings and resources. Mobile numbers were collected to identify duplicate participants and distribute e-gift cards. All the materials were provided in Korean, and responses were expected in Korean. Importantly, this study intentionally refrained from offering specific descriptions of multicultural populations to examine participants’ perceptions of ethnic diversity. Participants were fully informed of the research objectives and their right to voluntary participation before completing the survey.

### Research Instruments

The research instruments utilized in this study comprised a set of measures designed to assess various aspects of general hospital workers’ intercultural competences. As detailed in Appendix, the final survey questions employed four styles, including Likert scale responses ranging from one for ‘strongly disagree’ to five for ‘strongly agree,’ multiple-response question style, and a non-mandatory comment. The internal consistency of the survey items, measured by Cronbach’s alpha, was .862.

The first set of questions focused on demographic and background information, covering eight aspects including participants’ region, age, sex, occupation, years of experience, multicultural experiences (having foreign friends, overseas work experience, living abroad over six months), English competency, and the number of multicultural patients attended to per year.

For the second set of questions, we identified five domains in intercultural competence based on relevant literature that were applicable to the context of Korean general hospital workers. Cross-cultural awareness^27^^,^^29^^,^^36^^,^^45^ and knowledge^27^^,^^29^^,^^33^^,^^36^^,^^45^ assessed understanding relate to a comprehensive self-evaluation of one’s cultural and professional background, including attitudes towards multiculturalism, training and education experience, and medical knowledge regarding other cultures (Alpha = .78). Cultural sensitivity^29^^,^^30^^, ^^33^^, ^^46^ measured attitudes toward diverse cultures, considering one’s biases, cultural customs, and values (Alpha = .87). Intercultural communication skills^30^^,^^34^ evaluated language proficiency, empathy, and required time for medical treatment (Alpha = .61). Finally, self-efficacy^27^^,^^31^^,^^45^^,^^47^ assessed one’s self-belief in their ability to successfully treat multicultural patients (Alpha = .91).

In the final section, a non-mandatory comment section was included to afford participants the opportunity to provide additional comments if desired. To ensure the credibility and validity of all questionnaire items, the survey underwent a comprehensive review by experts before distribution.

### Data analysis

The collected data were comprehensively analyzed using SPSS/Win 27 to examine the intercultural competences of hospital workers. Given the survey’s structured format, participants selected predefined choices instead of providing open-ended responses.

Second, we carried out a detailed distribution analysis to investigate variations within these demographic categories and five domains of cross-cultural awareness and knowledge, cultural sensitivity, intercultural communications skills, and self-efficacy. To ensure consistency in the data-handling process, we processed certain reverse-coded questions to align them consistently with the Likert scale presentation. Thus, we first analyzed participant distribution among different demographic categories and computed sum scores for our dependent variables. For normally distributed data, we employed a one-way analysis of variance (ANOVA) with Bonferroni post-hoc analysis. In cases of non-normally distributed data, the study employed the Mann-Whitney U and Kruskal-Wallis tests. Nonparametric post-hoc analysis was performed using pairwise comparisons from the Kruskal-Wallis test results.

Finally, to gain a deeper understanding of the factors impacting intercultural competences, we conducted multiple linear regression analyses for independent variables showing significances in distribution analysis, including age, English competency, multicultural experience, multicultural training experience, occupation, organizational settings and resources. Categorical data were transformed into indicator variables using dummy variables, facilitating their inclusion in the regression analysis.

## Results

Regarding cross-cultural awareness (CCA), an overwhelming majority of participants, more than 85% (n=362), recognized the significance of multiculturalism within Korean society. They viewed it as essential for understanding the diverse cultures of patients, families, and healthcare providers. Additionally, over 60% (n=284) expressed the need for more multiculturalism-related content and training. About 61% (n=266) respondents perceived foreign and multicultural patients as beneficiaries of Korean medical care. The data showed that participants recognized the importance of multiculturalism in Korean society, with the majority of them acknowledging the medical benefits that multicultural patients receive from the Korean healthcare system. Additionally, the data revealed a lack of training and content related to multicultural topics in medical fields.

In relation to cross-cultural knowledge (CCK), slightly more than 50% (n=236) participants admitted to lacking knowledge about health behaviors, pregnancy/delivery customs, specific genetic-related habits, or familial impacts on patients’ medical decisions in other cultures. Approximately 15% (n=46) felt confident in understanding of health-related customs in different cultures. The participants exhibited limited knowledge of multicultural patients’ medical decisions, contrasting with a minority who expressed confidence in understanding health-related customs in different cultures.

For cultural sensitivity (CS), over 55% (n=282) of respondents (strongly) agreed on the importance of cultural sensitivity in the workplace. This included elements such as monitoring bias and respecting cultural and religious values. About 81.4% (n=330) emphasized respecting cultural and religious values. Approximately 56% (n=246) expressed a desire to learn about the cultural customs of others, and 75% (n = 329) considered understanding communication in diverse cultures crucial. Out of 439 respondents, a substantial majority of 70% (n=307) (strongly) agreed with the idea that foreign and multicultural patients should have a fundamental understanding of the Korean language and culture.  From the data, the respondents highlighted the importance of cultural sensitivity in the workplace, including aspects such as monitoring bias and respecting cultural and religious values. Additionally, a substantial majority underscored the need for multicultural patients to acquire knowledge of the Korean language and culture.

In terms of intercultural communication skills (ICS), 72% (n=315) participants encountered linguistic difficulties when treating foreign and multicultural patients. Furthermore, 80% (n=352) (strongly) agreed they needed more time for consultations with foreign and multicultural patients than Korean patients. From the data, it is evident that general hospital workers encountered significant linguistic difficulties when treating foreign and multicultural patients, which in turn necessitated more time for consultations.

Finally, regarding self-efficacy (SE), hospital workers expressed low self-efficacy in dealing with multicultural patients. About 11% (n=49) felt competent, while 44.2% (n = 194) felt incapable. They encountered challenges when setting up culturally suitable health service plans, with 58% (n = 253) finding it challenging. Additionally, 50% (n=218) disagreed with their ability to evaluate cultural characteristics comprehensively, and 44% (n=191) disagreed with providing information using patients’ cultural strengths. From the results, the participants demonstrated low compatibility in terms of self-efficacy when interacting with multicultural patients, with a significant percentage feeling incompetent and encountering challenges in setting up culturally suitable health service plans.

A non-mandatory comment section was provided for participants to freely share additional opinions or ideas in Korean. Out of 439 participants, 44 participants submitted additional comments. The comments were categorized into 12 groups as follows: the importance of translators, raising cross-cultural awareness, the necessity of multicultural education and training, suggesting differentiating the definition of multicultural patients (English vs. non-English speakers; insurance coverage levels; Korean-Chinese as foreigners, etc.), multicultural patients as beneficiaries, enhancing organizational settings and resources, raising cultural sensitivity in a workplace, awareness of multicultural children, reasons for hesitating to treat certain religious groups (e.g., Muslim foreigners), considering multicultural population as members of Korean society, and other survey comments (e.g., readability of the survey in mobile). The specific comments are not shown here to protect participants’ privacy and identification and will be analyzed in future studies.

Table 3 demonstrates the results of the group comparisons. Despite the respondents working in different hospitals, there were no significant inter- and intra-group differences for all dependent variables according to region. Regarding sex, CCA (U=12861.5, p < .001) and CS (U = 14658.5, p< .05) showed statistically significant differences. Regarding age, there were substantial differences in CCK (c^2^_(3)=_10.34, p< .05) and SE (c^2^(3) = 13.95, p<.01). Those in their 20s (Revised in accordance with the comment.) and 40s (p < .01) had higher CCK than those in their 30s, and those in their 20s (p < .01) and 40s (p < .01) also had higher SE than those in their 50s. Regarding occupation, there were significant differences in CCA, F_(5, 433)_ = 7.16, p< .001, and CS, F_(5, 433)_ = 7.68, p < .001. Hospital service workers (p< .01), healthcare providers (p < .001), and medical technicians (p<.001) had higher CCA than nursing assistants. In addition, healthcare providers (p<.001) and medical technicians (p<.01) had higher CS than nursing assistants.

In terms of multicultural experiences, all five dependent variables showed significance. In particular, the “no experience” group had lower CCA (c^2^_(3)_ = 26.82, p < .001) than the groups “have foreign friends (p< .01),” “overseas work experience (p< .01),” and “lived abroad (p < .001).” In CCK (c^2^_(3)_ = 10.64, p< .05), the “no experience” group had lower values than the “overseas work experience” group (p< .01). In addition, the “no experience” group had lower CS (c^2^_(3)_ = 12.27, p < .01) and SE (c^2^_(3)_ = 20.82, p< .001), respectively, than the “have foreign friends” group (p< .01; p < .01), the “overseas work experience” group (p<.05; p<.001), and the “lived abroad” group (p < .05; p < .05). Finally, the “no experience” group had lower ICS, F_(__3, 494)_ = 4.73, p < .01, than the “overseas work experience” group (p<.05) and the “lived abroad” group (p< .05).

As for English competency, there were statistical significances among all groups in CCA (F_(3, 435)_ = 11.91, p < .001), CCK (F_(3, 435)_ = 8.11, p < .001), CS (F_(3, 435)_ = 8.18, p < .001), ICS (F_(3, 435) _= 10.47, p < .001), and SE (F_(3, 435)_ = 13.67, p < .001). Among them, the “no English competency (none)” group had lower CCA compared to the intermediate (p < .001) and advanced (p<.001).  In CCK, the “none” group had lower knowledge than the intermediate group (p<.001). The “none” group also had lower CS than the beginner (p < .05), intermediate (p<.001), and advanced (p<.01) groups. Finally, the “none” group had lower ICS and SE than the intermediate (p < .01; p < .01) and advanced (p < .01; p < .001) groups.

There were differences regarding the number of foreign and multicultural patients attended to per year in CCA (c^2^_(3)_ = 18.28, p<.001), CCK (c^2^_(3)_ = 10.31, p< .05), CS (c^2^_(3) _= 13.46, p< .01), and SE (c^2^_(3)_ = 26.99, p< .001). Among them, the group of “no foreign and multicultural patients (no patients)” had substantially lower CCA than the group of “had 20 or more foreign and multicultural patients (p<.001).” Also, the “no patients” group had lower CCK than the groups of “had one to nine patients (p < .01)” and “had 10 to 19 patients (p<.01).” Finally, the “no patients” group also had lower CS and SE than “had one to nine patients (p < .05; p < .01),” “had 10 to 19 patients (p < .01; p < .001),” and “had more than 20 patients (p < .01; p < .001).”

Regarding individuals’ multicultural training experiences, there were significant differences in CCA (c^2^_(3)_ = 12.19, p<.01) and CCK (F_(__3, 456)_ = 17.71, p< .001). In particular, the group that “had in undergraduate course” had higher CCK than the “no experience” group (p<.05). Also, the groups “had professional development (p<.05; p < .001)” and “volunteer work (p < .01; p < .001)” had better CCA and more CCK. Additionally, when asked about the necessity of multicultural content and training, approximately 65% (n = 388) of the respondents (strongly) agreed.

Significant differences existed between the CCK (c^2^_(3)_ = 19.75, p<.001), CS (c^2^_(3)_ =21.63, p<.001), and SE (c^2^_(3)_ = 19.05, p<.001) groups in the survey questions on organizational settings and resources. Compared to the group of “no support,” the groups of “peer support (p < .001; p < .001; p < .001),” “organizational awareness of multicultural importance (p < .001; p < .001; p < .001),” and “translator(s) (p < .05; p < .01; p < .01)” had more CCK, better CS, and SE.

Regression analysis was performed to identify the underlying factors associated with the participants’ CCA, CCK, CS, ICS, and SE. Table 4 demonstrates all the results from the regression analysis. The Durbin-Watson statistics were between 1.91 and 2.09, which were close to the reference value of two; therefore, there was no autocorrelation. The tolerance value was between 0.51 and 0.94. The variance inflation factor (VIF) ranged from 1.13 to 1.77, i.e., below the reference value of 10. There was no multicollinearity between the independent variables. The independent variables with significant differences were presented in each model table.

The regression model for the factors influencing hospital workers’ CCA was statistically significant, with an explanatory power of 24.2% (adjR^2^ = .17, F_(__36, 402)_ = 3.57, p < .001).

**Table 3:** Statistical comparisons between the groups for dependent variables

Variable		CCA		CCK		CS		ICS		SE	
		M	SD	M	SD	M	SD	M	SD	M	SD
Region	Seoul	11.77	1.710	14.92	5.010	23.90	3.897	10.87	2.261	13.31	3.813
	Bucheon	11.18	1.758	14.65	4.601	23.30	2.984	11.45	1.779	13.55	3.655
	Cheonan	11.39	1.767	13.63	5.546	22.95	4.254	11.25	2.087	12.62	3.925
	Gumi	11.20	1.582	14.01	3.931	22.77	3.278	11.15	1.868	12.92	3.394
Sex^b^	Male^a^	11.89	1.761	14.18	5.352	23.85	3.751	11.07	1.976	13.59	4.066
	Female	11.22***	1.670	14.18	4.716	22.95*	3.686	11.22	2.023	12.85	3.593
Age	20~29	11.18	1.655	15.02††	5.328	23.21	3.505	11.28	1.877	13.62†	3.791
	30~39^a^	11.39	1.718	13.06	4.426	22.98	3.964	11.10	2.015	12.39	3.333
	40~49	11.42	1.717	14.70†	4.810	23.17	3.733	11.29	1.947	13.64††	3.829
	≥50	11.48	1.762	14.16	4.818	23.31	3.576	11.05	2.243	12.35	3.766
Occupation	Hospital service workers	11.15**	1.664	13.40	5.540	21.91	3.991	11.04	2.163	12.88	4.378
	Nursing assistants^a^	9.65	1.093	13.77	3.787	20.31	2.923	10.19	2.040	13.92	2.637
	Healthcare providers	11.59***	1.683	14.53	4.695	23.72***	3.487	11.37	1.947	13.02	3.668
	Medical technicians	11.56***	1.703	13.66	4.455	23.63**	3.748	10.72	2.004	13.13	3.035
	Pharmacists	11.00	1.225	15.40	8.820	25.00	3.162	11.60	2.302	10.40	1.517
	Hospital operation workers	10.80	2.168	11.20	4.438	20.40	4.278	10.60	1.140	12.40	4.561
Years of work experience	1~4 years	11.21	1.705	14.86	5.089	22.56	3.795	10.95	1.978	13.31	3.549
	5~9 years	11.15	1.647	13.85	5.009	23.96	3.723	11.28	2.146	12.66	3.614
	10~19 years	11.62	1.757	13.38	4.662	23.14	3.625	11.25	1.738	13.06	3.549
	≥ 20 years	11.51	1.843	14.48	4.696	23.15	3.674	11.26	2.155	12.96	4.017
English competency	None^a^	10.89	1.405	12.51	4.498	21.81	3.853	11.47	2.269	12.37	3.735
	Beginner	11.24	1.684	14.06	4.687	23.11*	3.486	11.41	1.858	12.60	3.445
	Intermediate	12.11***	1.786	16.09***	4.967	24.36***	3.867	10.39**	1.905	14.51**	3.726
	Advanced	13.20***	1.398	16.30	6.447	25.80**	3.765	8.90**	2.025	18.20***	4.077
Multicultural patients per year	0^a^	11.16	1.502	12.95	4.229	22.24	3.859	11.50	2.200	11.48	3.762
	1~9 patients	11.19	1.636	14.37	4.416	23.07†	3.564	11.17	1.824	13.01††	3.269
	10~19 patients	11.51	1.830	15.06††	5.673	23.83††	3.296	11.12	2.254	13.78†††	3.769
	≥ 20 patients	12.13†††	1.914	14.60††	5.875	24.12††	4.051	10.82	1.999	14.58†††	4.061
Multicultural experience	No experience^a^	11.16	1.502	12.95	4.229	22.24	3.859	11.50	2.200	11.48	3.762
	Have foreign friends	11.19††	1.636	14.37	4.416	23.07††	3.564	11.17	1.824	13.01††	3.269
	Overseas work experience	11.51†††	1.830	15.06††	5.673	23.83†	3.296	11.12*	2.254	13.78†††	3.769
	Lived abroad ≥ 6 months	12.13††	1.914	14.60	5.875	24.12†	4.051	10.82*	1.999	14.58†	4.061
Multicultural Training experience	No experience^a^	11.11	1.551	13.59	4.730	22.85	3.688	11.21	2.000	12.69	3.690
	Had in undergraduate course	11.72	1.852	15.52*	4.859	23.82	3.825	11.17	2.101	13.53	3.322
	Had professional development	12.64†	1.604	20.60***	5.138	25.33	3.374	10.67	2.380	16.13	4.502
	Volunteer work	12.08††	1.778	17.00***	5.336	23.89	4.181	10.66	2.109	14.96	4.059
Organizational settings and resources	No support^a^	11.21	1.561	12.95	4.925	22.12	3.883	11.11	1.926	11.95	3.682
	Peer support	11.77	1.793	15.42†††	5.166	24.17†††	3.702	11.00	2.151	13.98†††	4.087
	Organizational awareness	11.56	1.721	15.21†††	5.016	23.89†††	3.609	11.12	2.101	13.76†††	3.769
	Translator(s)	11.42	1.752	14.24†	4.597	23.45††	3.635	11.22	2.045	13.23††	3.648

Note: Statistically significant p values are indicated in bold compared to the control group a.

a Control group; b Mann-Whitney U Test; * One-way ANOVA followed by Bonferroni’ post hoc test (*p<.05, **p<.01, *** p<.001); † Kruskal-Wallis Test followed by Pairwise comparison (†p<.05, ††p<.01, ††† p<.001)

**Table 4:** Regression analysis of factors affecting CCA, CCK, CS, ICS and SE

Model	Unstandardized Coefficients		Standardized Coefficients	t	p	VIF
	B	SE	*β*			
(Constant)	11.271	0.267		42.165	0.000***	
Occupation (Hospital service workers)	-0.438	0.211	-0.100	-2.076	0.039*	1.229
Occupation (Nursing assistants)	-1.680	0.345	-0.232	-4.862	0.000***	1.207
English competency (Advanced)	1.660	0.560	0.145	2.964	0.003**	1.268
Multicultural experience (Lived abroad ≥ 6 months)	-1.012	0.483	-0.097	-2.095	0.037*	1.127
Multicultural training experience (Had professional development)	0.768	0.271	0.131	2.833	0.005**	1.129
OS (Peer support & OR)	0.819	0.368	0.111	2.224	0.027*	1.323
Cross-cultural Awareness: *F*= 3.567***, *R*^2^: .242, _adj_*R*^2^: .174, *Durbin-Watson*=1.972						
(Constant)	12.061	0.751		16.064	0.000***	
Age (20s)	2.142	0.645	0.177	3.319	0.001**	1.548
Age (40s)	1.607	0.570	0.153	2.819	0.005**	1.592
Occupation (Hospital operation workers)	-4.639	2.118	-0.101	-2.190	0.029*	1.162
English competency (No English competency)	-1.491	0.606	-0.118	-2.463	0.014*	1.245
English competency (Intermediate)	2.485	0.694	0.194	3.583	0.000***	1.584
Multicultural training experience (Had professional development)	5.979	1.854	0.143	3.226	0.001**	1.066
Multicultural training experience (Had in undergraduate & Volunteer work)	4.131	1.734	0.114	2.383	0.018*	1.237
Multicultural training experience (All experiences)	13.571	2.421	0.266	5.604	0.000***	1.218
OS (Organizational awareness of multicultural importance)	1.892	0.749	0.134	2.526	0.012*	1.524
OS (All)	1.622	0.772	0.114	2.100	0.036*	1.596
Cross-cultural Knowledge: *F*= 3.898***, *R*^2^: .259, _adj_*R*^2^: .192, *Durbin-Watson*=2.085						
(Constant)	22.281	0.599		37.194	0.000***	
Occupation (Hospital service workers)	-1.591	0.473	-0.167	-3.364	0.001**	1.229
Occupation (Nursing assistants)	-2.530	0.774	-0.161	-3.267	0.001**	1.207
Occupation (Hospital operation workers)	-3.674	1.690	-0.105	-2.175	0.030*	1.162
Multicultural training experience (Had in undergraduate)	1.230	0.608	0.102	2.024	0.044*	1.252
Cultural Sensitivity: *F*= 2.664***, *R*^2^: .193, _adj_*R*^2^: .120, *Durbin-Watson*=1.909						
(Constant)	11.557	0.330		34.978	0.000***	
Occupation (Nursing assistants)	-1.494	0.427	-0.175	-3.498	0.001**	1.207
Occupation (medical technicians)	-0.920	0.382	-0.119	-2.411	0.016*	1.170
English competency (Intermediate)	-1.051	0.305	-0.198	-3.444	0.001**	1.584
English competency (Advanced)	-2.500	0.692	-0.186	-3.611	0.000***	1.268
OS (Translators)	0.604	0.289	0.124	2.087	0.038*	1.701
Intercultural Communication Skills: *F*= 2.156***, *R*^2^: .162, _adj_*R*^2^: .087, *Durbin-Watson*=2.089						
(Constant)	10.068	0.572		17.593	0.000***	
Age (20s)	1.311	0.492	0.142	2.666	0.008**	1.548
Age (40s)	1.093	0.434	0.136	2.516	0.012*	1.592
Occupation (Nursing assistants)	2.034	0.740	0.129	2.750	0.006**	1.207
English competency (Intermediate)	1.693	0.529	0.173	3.203	0.001**	1.584
English competency (Advanced)	4.870	1.199	0.196	4.061	0.000***	1.268
Multicultural patients attended to per year (1~9)	0.917	0.446	0.123	2.056	0.040*	1.961
Multicultural patients attended to per year (10~19)	1.361	0.576	0.130	2.365	0.018*	1.656
Multicultural patients attended to per year (≥20)	1.698	0.588	0.165	2.886	0.004**	1.773
Multicultural training experience (Volunteer work)	1.212	0.580	0.095	2.089	0.037*	1.129
Multicultural training experience (All)	4.348	1.845	0.111	2.356	0.019*	1.218
OS (Organizational awareness of multicultural importance)	1.221	0.571	0.113	2.138	0.033*	1.524
OS (All)	1.777	0.588	0.163	3.020	0.003**	1.596
Self-efficacy: *F*= 3.968***, *R*^2^: .262, _adj_*R*^2^: .196, *Durbin-Watson*=2.002						

*OS: Organizational settings and resources; OR: Organizational awareness of multicultural importance (*p<.05, **p<.01, *** p<.001).

Significant influencing factors with positive correlations were an advanced level of English competency (ß= .15, p < .01), multicultural training experience in professional development (ß=.13, p<.01), and peer support with organizational awareness of multicultural importance in organizational settings and resources (ß =.11, p<.05). On the contrary, significant influencing factors with negative correlations were occupations with hospital service workers (ß = -.10, p < .05) and nursing assistants (ß=-.23, p<.001) and multicultural experience with six more months lived abroad (ß = -.10, p< .01).

The regression model for the factors influencing hospital workers’ CCK was statistically significant, with an explanatory power of 25.9% (adjR^2^ = .19, F_(__36, 402) _= 3.90, p< .001). The positive influencing factors were those in their 20s (ß = .18, p < .01) and 40s (ß = .15, p < .01), having an intermediate level of English competency (ß = .19, p < .001), multicultural training experiences with professional development (ß=.14, p < .01), undergraduate education and volunteer work (ß = .11, p<.05), and all three experiences of undergraduate education, professional development, and volunteer work (ß=.27, p< .001). Also, there were positive influencing factors in all three organizational settings and resources, including awareness of multicultural importance, peer support, and translators (ß = .11, p<.05). In contrast, there were only two independent variables with negative influences: hospital operation workers (ß = -.10, p<.05) and having no English competency (ß= -.12, p<.05).

The regression model for the factors influencing hospital workers’ CS was statistically significant, with an explanatory power of 19.3% (adjR^2 ^= .19, F_(__36, 402)_ = 2.66, p<.001). There was only one positive influencing factor: multicultural training experience during undergraduate education (ß = .10, p < .05). On the contrary, negative correlation factors were occupations associated with hospital service workers (ß = -.17, p < .01), nursing assistants (ß = -.16, p < .01), and hospital operation workers (ß = -.11, p < .05).

The regression model for the factors influencing hospital workers’ ICS was statistically significant, with an explanatory power of 16.2% (adjR^2^ = .16, F_(__36, 402)_ = 2.16, p < .001). There was only one positive factor: translators (ß = .12, p < .05). In contrast, negatively significant influencing factors were occupations with nursing assistants (ß = -.18, p < .01) and medical technicians (ß = -.12, p < .05), and English competencies at intermediate (ß = -.20, p < .01) and advanced levels (ß = -.19, p < .001).

The regression model for the factors influencing hospital workers’ SE was statistically significant, with an explanatory power of 26.2% (adjR^2 ^= .20, F_(__36, 402) _= 3.97, p < .001). There were no negative influencing factors. On the contrary, significant influencing factors with positive correlations were the age groups of the 20s (ß = .14, p < .01) and 40s (ß = .14, p < .05), nursing assistants (ß = .13, p < .01), and English competency levels of intermediate (ß = .17, p < .01) and advanced (ß = .20, p < .001). Also, the groups having multicultural patients per year showed correlations with SE: patients of one to (ß = .12, p < .05), 10 to 19 (ß = .13, p < .05), and more than 20 (ß = .17, p < .01). Furthermore, it was statistically significant for one’s SE to have multicultural training experiences in volunteer work (ß = .10, p < .05) and have all undergraduate education, professional development, and volunteer work (ß = .11, p < .05). Finally, all three organizational settings and resources, organizational awareness, peer support, and translators (ß = .16, p < .01), correlated with SE.

## Discussion

The findings of this study underscored that the participants recognized the importance of multiculturalism in Korean society, acknowledging medical benefits for multicultural patients within the Korean healthcare system. Although the study found that participants acknowledged the significance of multiculturalism, it also revealed a notable lack of multicultural training in medical fields. This deficiency in training was reflected in their limited knowledge of health behaviors and customs affecting multicultural patients, highlighting the critical need for cultural sensitivity and language proficiency among hospital workers. Linguistic barriers and low self-efficacy further compounded the challenges in providing culturally appropriate healthcare services.

The inferential statistical analyses revealed significant disparities in intercultural competence domains among different demographic and background groups of general hospital workers in Korea. Various factors were found to influence different domains of intercultural competence among hospital workers. Notably, factors such as sex, occupation, the number of foreign and multicultural patients attended to per year, and multicultural training experiences significantly impacted cross-cultural awareness, while age, the number of foreign and multicultural patients attended to per year, multicultural training experiences, and organizational settings and resources played crucial roles in determining cross-cultural knowledge. Intercultural communication skills were substantially impacted by linguistic difficulties. Cultural sensitivity was notably affected by factors such as sex, occupation, and organizational settings, while age and organizational resources were major determinants of self-efficacy. Overall, the inferential statistical analyses shed light on the complex interplay of factors influencing intercultural competence among hospital workers and emphasize the significance of tailored approaches to foster intercultural competence in healthcare settings.

The multiple regression analysis revealed crucial factors affecting hospital workers’ intercultural competences emerged. Advanced English competency, multicultural training experiences in professional development, and peer support with organizational awareness of multicultural importance were significant positive contributors to intercultural competence. Conversely, occupations such as hospital service workers and nursing assistants, as well as limited multicultural experiences, were associated with lower levels of intercultural competence.

These findings underscore the significance of targeted interventions, such as language training and multicultural education programs, to enhance hospital workers’ intercultural competences and improve the quality of care for foreign and multicultural patient populations. In conclusion, our study verifies that English proficiency, multicultural experience, multicultural training experiences, and organizational settings and resources emerge as the most crucial factors influencing the development of all five intercultural competences across all groups.

Notably, the participants who did not have multicultural experience or English proficiency demonstrated considerably lower levels of all intercultural competences than other groups, particularly those with intermediate or advanced English proficiency. Portillo and colleagues^39^ research emphasizes that patients’ limited English proficiency could be a risk factor for health care, so improving the overall understanding of the language barrier can positively impact clinical care. The results of this study also highlighted that limited cultural experience and poor English proficiency can obstruct the proper care of multicultural patients in general hospitals. Given its pivotal role, solid English proficiency is crucial for hospital workers, as it equips them with the necessary intercultural competence as shown in previous studies.

Second, it was evident that peer support and awareness of multicultural topics at an organizational level could contribute to heightening cross-cultural awareness and communication among general hospital workers. Comparing with the existing literature, previous studies emphasize that establishing comprehensive organizational settings and resources is vital for enhancing cross-cultural knowledge. In particular, ensuring culturally safe environments, as well as the presence of translators within an organization, proves crucial for fostering intercultural communication skills to improve hospital workers’ emotional and mental security, which is fundamental for effectively treating multicultural patients.^37^^,^^48^ Acknowledging its critical importance, it is imperative to establish comprehensive organizational settings and resources, including language support services (e.g., translators) in general hospitals, to foster intercultural communication skills among hospital workers, as highlighted by previous studies.

Finally, it was conspicuous that the participants lacking multicultural experience demonstrated significantly lower levels of all intercultural competences compared to other groups. Nevertheless, acquiring multicultural experience (e.g., having foreign friends or living abroad) presents inherent complexities and challenges. In contrast, multicultural training can offer a structured approach to acquiring essential knowledge, indirect exposure, and building intercultural competences. The studies by Schenk and colleagues,^37^ Osmancevic and colleagues,^49^ Schouten and colleagues,^38^ Paternotte and colleagues,^50^ and Hudelson and colleagues,^51^ collectively underscore the vital role of intercultural education and training in healthcare. Previous research emphasizes that factors such as staff diversity and training significantly impact healthcare workers’ cultural competence, emphasizing the need for comprehensive intercultural training programs. Furthermore, they claim that installing and merging multicultural education and training tracks into medical education is indispensable for healthcare professionals to effectively address diverse patients’ needs, ultimately leading to more patient-centered and culturally sensitive care. Conceding its utmost significance, investing in comprehensive intercultural training programs is imperative to equip hospital workers with the necessary skills and competences to provide patient-centered and culturally sensitive care. In conclusion, investing in comprehensive intercultural training programs is imperative to equip hospital workers with the necessary skills and competences to provide patient-centered and culturally sensitive care and to build culturally inclusive organizational environment.

This study contributes to the existing literature by shedding light on the multifaceted nature of intercultural competence among hospital workers and advocating for tailored interventions to address the identified gaps effectively. However, the study still has several limitations. Firstly, the use of self-administered survey questions may introduce challenges related to respondent comprehension, motivation, and social desirability bias in providing answers. Secondly, a more balanced distribution of participants from diverse occupational groups is necessary to ensure a comprehensive representation. Thirdly, the study’s explanatory power concerning factors influencing intercultural competences is limited due to the diversity of surveyed occupations. Lastly, the limited number of questions evaluating intercultural communication skills may have resulted in low reliability. Therefore, future study should consider a specific occupational group within general hospitals with more questions related to intercultural communication skills could provide more in-depth insights into hospital workers’ intercultural competences.

## Conclusions

Based on the research conducted, this study aimed to assess the current level of intercultural competence among Korean general hospital workers, identify influencing factors, and propose strategies for enhancing their overall intercultural competence within healthcare environments. The findings highlighted the significance of English proficiency, multicultural experience, and organizational settings in shaping the intercultural competences of hospital workers across various domains such as cross-cultural awareness and knowledge, cultural sensitivity, intercultural communication skills, and self-efficacy.

To enhance intercultural competences among general hospital workers, several key recommendations are proposed. Firstly, the establishment of resident translators within hospitals is deemed crucial to overcome linguistic barriers and facilitate effective communication with multicultural patients. While English proficiency remains essential, providing translators can significantly enhance communication and patient care.

Secondly, organizing information sessions aimed at providing medical information to multicultural patients can foster mutual understanding between hospital workers and multicultural patients. This approach can enable patients to express concerns or worries regarding medical procedures, shifting the focus from considering multicultural patients solely as beneficiaries to engaging them as active participants in their healthcare journey.

Thirdly and most importantly, improving hospital workers’ intercultural competences through targeted medical education, professional development, and training tracks related to cultural diversity is vital. Based on our findings, a tri-tiered training approach is recommended as viable substitutes for personal multicultural experiences, aiming to enhance the intercultural competences of general hospital workers and ultimately improve patient-centered and culturally sensitive care within Korean healthcare environments.

The first tier involves implementing multicultural training for all employees to build a shared understanding of multiculturalism. This step can include multicultural collective training, facilitating an organizational understanding of diversity in medicine. This shared-knowledge is crucial for providing patient-centered care that respects diverse cultural backgrounds.

The second tier comprises on occupation-specific courses for practical cross-cultural knowledge and cultural sensitivity. During this stage, short-term training courses for each occupation will allow hospital workers to practice their cross-cultural knowledge and examine coworkers with cultural sensitivity, utilizing role-plays and situational response practices. This hands-on approach allows them to develop a deeper understanding of patients with diverse cultural backgrounds, leading to more culturally sensitive care.

Finally, the last tier focuses on long-term managerial programs to prepare managers and executives to effectively lead in culturally diverse healthcare settings. By providing ongoing professional development for managerial-level general hospital workers, the program ensures that leadership is equipped to address challenges related to cultural diversity and implement strategies for improving patient-centered care. This tier emphasizes the importance of continuous learning and adaptation to future changes in healthcare environments. Overall, this tri-tiered training approach offers a comprehensive strategy to enhance intercultural competences among hospital workers, fostering a culture of inclusivity and improving healthcare outcomes for diverse patient populations.

### Acknowledgements

This work was supported by the Soonchunhyang University Research Fund. We would also like to express our gratitude to the Soonchunhyang University Gumi Hospital Professor Association of Research and Professor Hun-Gyu Hwang for his thorough feedback and support. Their unwavering support and collaboration have significantly contributed to the advancement of scientific knowledge in this study.

### Conflicts of Interest

The authors declare they have no conflicts of interest.

## Supplementary material

Supplementary file 1 Appendix. Survey Questionnaire
