# Supplementary material for: Factors influencing intercultural competences of hospital workers for multicultural patients in South Korea
Source: Int J Med Educ. 2024 Jun 21;15:66–79. doi: 10.5116/ijme.6667.2270 (PMC11285027; doi:10.5116/ijme.6667.2270)
Supplement: Supplementary file 1 — Appendix. Survey Questionnaire [file ijme-15-66-S1.pdf]

Appendix

Survey Questionnaire

Category	No.	Question
Demographic Questions	0	Regions: ①Seoul, ②Gumi, ③Cheonan, ④Bucheon
	1	Sex: ①Male, ②Female
	2	Age (Write down)
	3	Occupation: ①Hospital service workers ②Nursing assistants ③Healthcare providers ④Medical technicians ⑤Pharmacists ⑥Hospital operation workers ⑦ETC: Write down
	4	Years of work experience: ①1 year~5 years, ②5 years~10 years, ③10 years~20 years, ④above 20 years
	5**	Multicultural experiences: ① No experience, ②Have foreign friends, ③Overseas work experience, ④Lived abroad ≥ 6 months
	6	English competency: ①None at all, ②Beginner, ③Intermediate, ④Advanced
	7	Multicultural patients per year: ①0, ②1~9 patients, ③10~19 patients, ④≥ 20 patients
Cross-Cultural Awareness	8	Multiculturalism is becoming increasingly important in Korean society.
	9	Patients, caregivers, medical officials, etc., should know each other's cultural background.
	10	I am sensitive to prejudices related to foreign/multicultural patients.
	11	More multicultural content and training related to foreign/multicultural patients are needed.
	12*	Foreign/multicultural patients are beneficiaries of various benefits of Korean medical care.
	13**	Asking about receiving multicultural-related training, professional development, undergraduate education, and/or volunteer work experience
Cross-Cultural Knowledge	14	I know about other cultural areas' health customs (health behaviors).
	15	I know about faith and customs concerning death in other cultural contexts.
	16	I know about health beliefs in other cultural areas.
	17	I know about faith and customs concerning pregnancy and delivery in other cultural areas.
	18	I know about specific (genetic) diseases common in other cultural areas.
	19	I know how the patient's family influences decision-making in each culture when the patient makes health-related decisions.
	20**	Asking about organizational settings and resources (peer support, equipping translators, and/or having an organizational awareness of multicultural importance pertinent to multicultural patients)
Cultural Sensitivity	21	As a healthcare service provider, I think I have to check my bias toward race or culture, which may affect my behavior.
	22	I think it is essential to consider the patients' demographics (social and economic status) and characteristics such as age, gender, and religion.
	23	I want to learn about the cultural customs of various countries.
	24	I respect various cultural values.
	25	I think it is important to consider religious differences.
	26	I think it is essential to consider the ways other cultures express themselves.
	27	I think foreign/multicultural patients should have minimal language acquisition and cultural knowledge to receive health-related services in South Korea.
Intercultural Communication Skills	28*	I have linguistic difficulties treating foreign/multicultural patients.
	29*	I need more time than Korean patients when I treat foreign/multicultural patients.
	30*	It is embarrassing for me when foreign/multicultural patients share their health/treatment plans.
Self-efficacy	31	I am competent in dealing with foreign/multicultural patients.
	32	I can set up a health-related service plan suitable for each culture.
	33	I can accept patients' cultural backgrounds and provide services that meet their medical needs.
	34	I can provide information or help by using each patient's cultural strengths.
	35	I can comprehensively evaluate the cultural characteristics of multicultural patients.
Non-mandatory Comments	36	Feel free to write down any thoughts or suggestions you want to share about this study.

* Reverse questions
**Multiple response question
